# 0548. Effects of sedation and muscle paralysis on inflammation during mechanical ventilation

**DOI:** 10.1186/2197-425X-2-S1-P32

**Published:** 2014-09-26

**Authors:** LG Ayala, M Abreu, M Avila, BC Bergamini, AC Neto, WA Zin, A Giannella Neto, AR Carvalho

**Affiliations:** Federal University of Rio de Janeiro, Laboratory of Pulmonary Engineering, Rio de Janeiro, Brazil; Federal University of Rio de Janeiro, Carlos Chagas Filho Institute of Biophysics, Rio de Janeiro, Brazil

## Introduction

A significant reduction in mortality was observed in severe ARDS patients when neuromuscular blockage (NMB) was used in the acute phase (Papazian L. et al, 2010).

## Objectives

We aim to evaluate pro- and anti-inflammatory markers immediately after two hours ventilation in lung-injured rats with different sedation protocols.

## Methods

Thirty male Wistar rats were divided into five groups (6 animals each): low sedation, high sedation (with or without NMB) and a healthy, non-ventilated control group. Animals were anesthetized with 2.8 or 1.4%vol isoflurane and 2.5 or 0.5 mg/kg midazolam in high and low sedation groups, respectively; and 10 mg/kg +10 mg/kg/h atracurium in NMB group. After intubation and stabilization period (baseline settings: VT= 8 mL/kg, FR=70 rpm, FiO2=50%, PEEP=3, I:E = 1:2), lung injury was induced by intratracheal injection of LPS (15 mg/kg) + ventilation induced lung injury (2.5xVT, FR=32, PEEP=10 cmH_2_O for 45s) (Dixon et al, 2009). Thereafter animals were ventilated for 2 hours with baseline settings. Airway (Paw), esophagic (Peso) were continuously recorded (200 Hz sampling frequency). The mean power of Peso was then calculated, by means of the spectral density estimation (Welch's periodograms, 400 samples window, 50% overlap). At the end of ventilation, mean arterial pressure (MAP) and gas analysis were evaluated in all ventilated groups. Lungs were extracted for cytokines (IL-6, IL-10) measurement. Euthanasia was performed by exsanguination associated to isoflurane overdose. Data were compared with Shapiro-Wilk, ANOVA and Bonferroni post-hoc tests, p ≤ 0.05.

## Results

No differences in mean MAP and heart rate were observed. PaO_2_/FiO_2_ and PCO_2_ were higher in non-paralized groups compared to paralyzed groups (p=0.01). Peak and mean Paw were significantly lower (p≤0.01) whereas the mean power of Peso was higher (p=0.004) in the low sedation without NMB. IL-6 concentration was also significantly lower in this group (p< 0.001) and presented a negative correlation (r = -0.59, R^2^=0.34 and p= 0.002) with Mean power of Peso. Furthermore, IL-10 concentration was higher in the low sedation without NMB compared to the others (p=0.002) and also correlates with the mean power of Peso (r = 0.60, R^2^= 0.36 and P = 0.002).

## Conclusions

Animals with a superficial sedation scheme in absence NMB might have more recruited lungs (lower Ppeak and Pmean), less inflammation and higher anti-inflammatory markers. The long-term consequences must be further evaluated.
